# Pulsed Doppler A-wave as an aid in patient selection for atrioventricular synchrony through a leadless ventricular pacemaker

**DOI:** 10.1007/s10840-022-01288-9

**Published:** 2022-07-04

**Authors:** Margarida Pujol-López, Cora Garcia-Ribas, Adelina Doltra, Eduard Guasch, Sara Vazquez-Calvo, Mireia Niebla, Rebeca Domingo, Ivo Roca-Luque, José M. Tolosana, Lluís Mont

**Affiliations:** 1 Institut Clínic Cardiovascular (ICCV), Hospital Clínic, Universitat de Barcelona, C/Villarroel 170, 08036 Barcelona, Catalonia Spain; 2 Institut d’Investigacions Biomèdiques August Pi i Sunyer (IDIBAPS), Barcelona, Catalonia Spain; 3 Centro de Investigación Biomédica en Red Enfermedades Cardiovasculares (CIBERCV), Madrid, Spain

Micra AV (Medtronic, Minneapolis, USA) is a ventricular leadless pacemaker that maintains atrioventricular (AV) synchrony by sensing the atrial mechanical signal (A4-atrial kick). The MARVEL 2 (*Micra Atrial TRacking Using a Ventricular AccELerometer*) algorithm has demonstrated a high proportion of AV synchrony in patients with AV block (95% had ≥ 70% AV synchrony) [1]. An E/A ratio < 0.94 combined with low sinus rate variability has been suggested to indicate a 90% probability of correct A4 amplitude and high AV synchrony [2]. However, the usefulness of echocardiography in the selection of Micra AV candidates has not been established.

Our study analyzed whether echocardiographic characteristics of the atrium could be used to predict correct AV synchrony in a leadless VDD pacemaker. The hypothesis was that the A-wave velocity determined by Doppler echocardiography could be used to predict the appropriate atrial sensing in patients with a leadless VDD pacemaker indication.

Patients who received a leadless Micra AV implant from July 2020 to April 2021 at our institution were included. An echocardiogram was performed at baseline and 1 month after implantation. All studies were performed and reviewed by 2 experienced echocardiographers (CGR and AD). Left atrial volumes, mitral Doppler patterns (E and A waves), and *strain* curves were analyzed. Left atrial volumes and functional left atrial parameters have been previously associated with loss of atrial sensing in VDD pacemakers [3]. Atrial tracking was evaluated by using device interrogation and checking the percentages of appropriate atrial sensing in the cumulative frequency histograms. All patients signed an informed consent form for the study.

Eight consecutive patients in sinus rhythm (50% women, mean age 68 years) were included. Of these patients, 75% had complete AV block, and 25% had 2:1 AV block. Three patients were on immunosuppressive drugs, 2 had no venous access (one on hemodialysis), and 3 had a previous infection (endocarditis that required surgery or device pocket infection).

The device was implanted without complications in all patients. The mean threshold was 0.67 V (0.24 ms), and the mean impedance was 893 ohms; in all but 1 patient, the device was deployed at the first position.

**Fig. 1:**
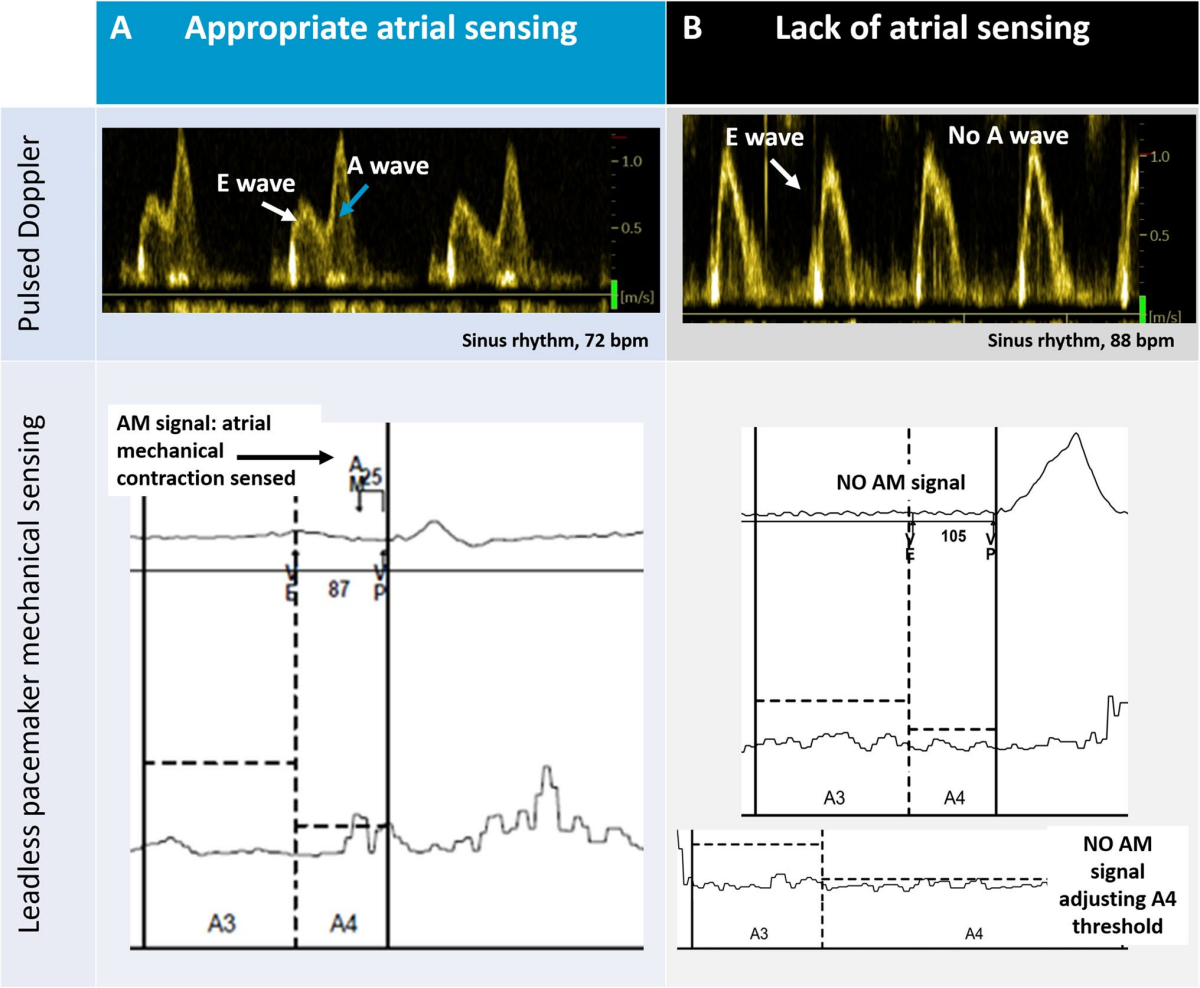
Atrial mechanical contraction detected with pulsed Doppler. Panel **A** shows a patient with an A-wave of 122 cm/s and correct atrial mechanical (AM) sensing by the device. In Panel **B**, the patient is in sinus rhythm with the absence of an A-wave in the pulsed Doppler. AM atrial mechanical, bpm beats per minute

At a mean follow-up of 7 months (3 to 14 months), the AV algorithm provided AV synchrony in 6 (75%) patients (Fig. 1A). However, in 2 patients (25%), despite device checks and adjustments, the device could not sense the atrial kick in sinus rhythm. Both patients were programmed in VVIR mode.

The 2 patients in whom atrial mechanical sensing failed showed (a) a severely dilated atrium (85 ml in patient 1 and 175 ml in patient 2) and (b) a small A-wave (62 cm/s) or the absence of an A-wave (Fig. 1B). On the other hand, patients with appropriate atrial sensing had a less dilated atrium (mean atrial volume of 63 ml) and presented larger A-waves on pulsed Doppler (mean velocity of 125 cm/s vs. 31.5 cm/s; *p* < 0.05, nonparametric Mann–Whitney test). In this small series, a cutoff point of 73 cm/s for the A-wave velocity had 100% sensitivity and specificity (*p* < 0.05; area under the *receiver operating characteristic curve* of 1) for identifying patients with appropriate atrial sensing.

In a previous study, an E/A ratio < 0.94 predicted high AV synchrony in a multivariable model [2]. Fifty percent of our patients with appropriate A4 sensing showed an E/A ratio > 0.94; thus, in our small cohort, the proposed E/A ratio would not have correctly predicted postimplantation AV synchrony. Furthermore, *strain* curves could not be used to predict AV synchrony in our series. In one of the patients with poor sensing, the *strain* curves could not be analyzed due to acoustic shadow secondary to mechanical mitral valve. Our study is hypothesis generating; additional studies are necessary to assess the predictive value of pulsed A-wave for AV synchrony.

Lack of analysis of tricuspid Doppler flow is a limitation of the study. However, the E/A ratio and mitral Doppler patterns are much more commonly used parameters in clinical practice. Therefore, any echocardiography performed at any center could guide the decision regarding whether a patient is a good leadless candidate.

An A-wave velocity > 73 cm/s could predict appropriate atrial sensing by the Micra AV device. Performing echocardiography before pacemaker implantation could contribute to better selection of Micra AV candidates.
